# Supplementary material for: Low-noise optomechanical single phonon-photon conversion for quantum networks
Source: Nat Commun. 2026 Jan 5;17:1187. doi: 10.1038/s41467-025-67956-2 (PMC12859002; doi:10.1038/s41467-025-67956-2)
Supplement: Supplementary file 1 — Supplementary Information [file 41467_2025_67956_MOESM1_ESM.pdf]

Low-noise Optomechanical Single Phonon-photon Conversion for Quantum Networks

Liu Chen,[1,†] Alexander Rolf Korsch,[1,2,3,†] Cauê Moreno Kersul,[4] Rodrigo Benevides,[4] Yong Yu,[1] Thiago P. Mayer Alegre,[4] and Simon Gröblacher[1,*]

[1] *Kavli Institute of Nanoscience, Department of Quantum Nanoscience, Delft University of Technology, 2628CJ Delft, The Netherlands*
[2] *Department of Physics, Fudan University, Shanghai 200433, P.R. China*
[3] *Department of Physics, School of Science, Westlake University, Hangzhou 310030, P.R. China*
[4] *Instituto de Física Gleb Wataghin, Universidade Estadual de Campinas (UNICAMP), 13083-859 Campinas, SP, Brazil*

A. Finite-element simulations

The quasi-two-dimensional (2D) OMCs used in our work were first introduced in [1] and later optimized in [2, 3]. The general idea is to incorporate a line defect of C-shaped holes replacing the snowflake lattice [2]. With careful design, such structures can realize co-localization of both optical and mechanical modes. The effective mirror unit cell consists of both snowflakes and C-shape holes in the center. Figure S1 shows the finite-element simulation of such a mirror unit cell when Floquet periodic boundary conditions are applied. The pink shaded areas in Fig. S1b and d indicate the photonic and phononic bandgaps, extending from 170.8 THz to 203 THz, and from 9.99 GHz to 10.49 GHz, respectively. The black ticks indicate the simulated optical resonance with $\omega_c/2\pi = 195.02$ THz and mechanical resonance with $\omega_m/2\pi = 10.18$ GHz for the whole device, both of which are inside the bandgaps. The solid blue lines at the bandgap edges indicate the modes which mainly live in the C-shape region as our modes of interest. Specifically for the phononic modes, only modes with symmetry group $(\sigma_z = +1, \sigma_y = +1)$ couple strongly to the optical mode of interest.

Similar to 1D OMCs, a defect region is created by adiabatically tapering the parameters of the C-shape holes. The whole defect region consists of $7 \times 2 = 14$ cells. The transition function used between the mirror unit cell and defect center cell is [3]:

$$y_n = y_{\min} + (y_{\max} - y_{\min}) \cdot e^{-\frac{9\cdot(n-n_{\mathrm{def}})^2}{2\cdot n_{\mathrm{def}}^2}} \tag{S1}$$

where n is the index of the defect cell ranging from 7 to 1, $n_{\mathrm{def}} = 7$ represents the number of cells in half of the defect region. The defect is created by specifically changing parameters l_{arm}, l_{pad}, and w_{arm}, w_{pad}. In the transition function above, $y_{\max}$ represents the values of said parameters in the mirror cell ($n = 7$) and $y_{\min}$ represent the values in the defect center cell ($n = 1$).

The remaining design parameters are provided below in Table I:

Supplementary Table I. Design Parameters

Parameters	Value (nm)
h_{cell}	721
h_{snow}	435
l_{wav}	171.5
$l_{\mathrm{arm,max}}$	203
$l_{\mathrm{arm,min}}$	191.6
$w_{\mathrm{arm,max}}$	104
$w_{\mathrm{arm,min}}$	82
$l_{\mathrm{pad,max}}$	112
$l_{\mathrm{pad,min}}$	108
$w_{\mathrm{pad,max}}$	188
$w_{\mathrm{pad,min}}$	168
r_1	40
r_2	25

Alternative designs for two-dimensional OMC structures have recently been demonstrated [4]. The choice of the "boomerang" unit cell in [4] was mainly motivated by bringing down the mechanical frequency (∼7.4 GHz) to reduce the complexity of coupling to superconducting circuits. In addition, the boomerang unit cell has a larger filling factor (74.5%) than the snowflake unit cells (64.1%) [4], which could be beneficial for better thermalization. However, this is at the cost of smaller optical and mechanical band gaps from the waveguide unit cell, which are 27 THz wide for optical band gap and 0.15 GHz wide for mechanical band gap, compared to 32 THz optical and 0.5 GHz mechanical band gaps for the snowflake + Cshape unit cell used in our work. Moreover, the mechanical band of interest in [4] is very flat compared to the snowflake + Cshape structure, which makes the former more susceptible to fabrication imperfections [2].

B. Optical setup

Figure S2a shows the full experimental setup used to perform pulsed optomechanical measurements. Two tunable continuous-wave external diode lasers are locked to the blue and red optomechanical sideband of the optical cavity respectively. Both lasers are stabilized by active feedback using a wavemeter. We note that our laser stabilization is performed in an open-loop configuration with respect to the optical resonance of the OMC. This is

† These authors contributed equally to this work
* s.groeblacher@tudelft.nl

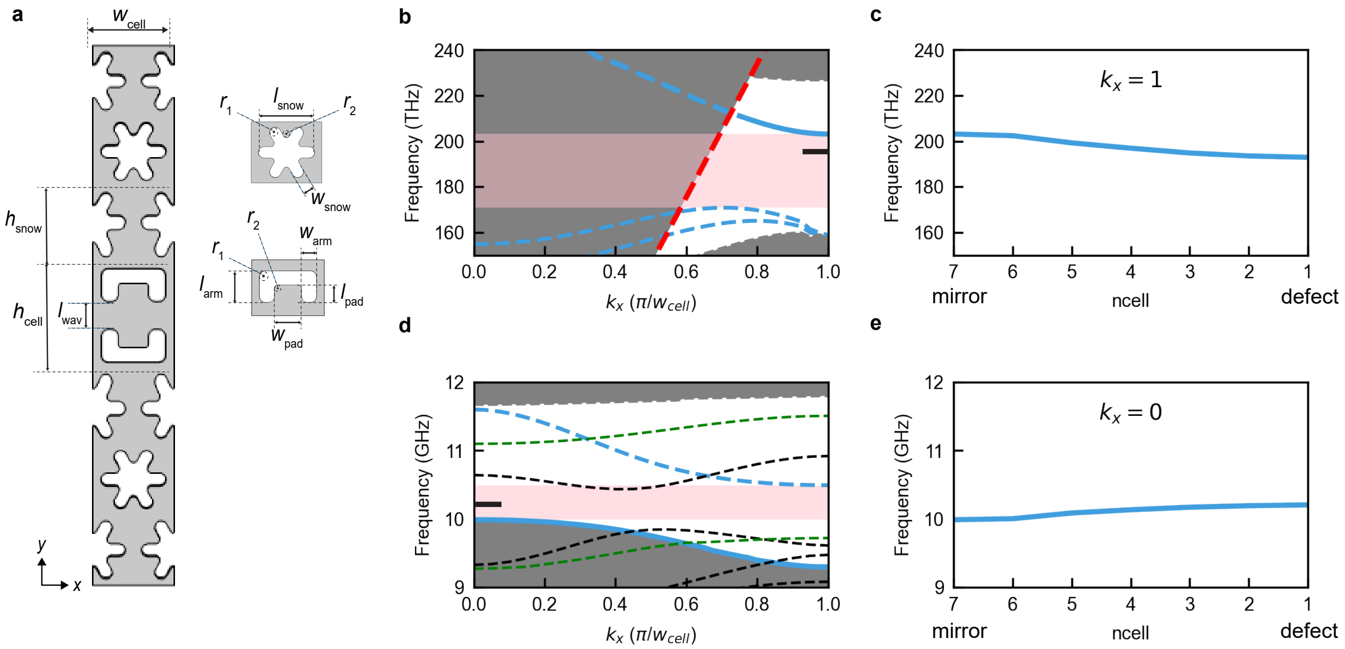

FIG. S1. **Finite element simulation of the unit cell.** **a** Schematic of the quasi-2D optomechanical crystal mirror unit cell consisting of both snowflakes and C-shape holes, $w_{\rm cell} = 502\,{\rm nm}$ is the unit cell width along x direction. The parameters for mirror unit cells are: $(l_{\rm snow}, w_{\rm snow}) = (201, 80.4)$ nm, $(l_{\rm arm}, l_{\rm pad}, w_{\rm arm}, w_{\rm pad}) = (203, 112, 104, 188)$ nm. For the rows of snowflakes which are closest to C-shapes, the length of the second half of the snowflake holes along y direction is scaled by 1.2. **b** and **d** show the photonic and phononic band diagrams of the mirror unit cell, respectively. The shaded grey areas represent modes of continuum. In the photonic band diagram, only transverse-electric (TE) modes are shown since they are predominantly guided in the silicon slab with a thickness of 250 nm. The dashed red line represents the light cone. The solid blue lines on the band gap edges are the C-shape modes of interest. The dashed blue lines show other guided modes in the structure. In the phononic band diagram, the dashed black lines are for modes with symmetry group $\sigma_z = -1$, the dashed green lines are for modes with symmetry group $(\sigma_z = +1, \sigma_y = -1)$. The pink shaded areas indicate the optical and phononic band gaps formed from such mirror cells. The black ticks indicate the optical and mechanical frequency of the whole device, which are inside the band gaps. **c** and **d** indicate how the band edge modes of interest shift in frequency when we change the parameters from mirror cell (index $ncell = 7$) to defect center cell (index $ncell = 1$).

possible because of the relatively broad optical linewidth $\kappa/2\pi = 2.4\,{\rm GHz}$ and the consequently very stable optical resonance frequency. We did not observe any significant drifts of the optical cavity resonance frequency over the measurement time of three months.

The light from both lasers is filtered using one fiber-coupled filter cavity (50 MHz linewidth) to suppress Gigahertz frequency laser noise. Pulses are created using 110 MHz acousto-optic modulators (AOMs) gated by a P400 pulse generator. The two paths for pulse generation are combined using a fiber beam splitter and routed to the OMC device inside the dilution refrigerator at base temperature $T = 20\,{\rm mK}$ via a fiber-based circulator. The light coming back from the device is filtered using three consecutive home-built free-space Fabry-Pérot cavities (each with linewidth 150 MHz) [5]. The filter cavities are locked to the frequency of the optical cavity of the OMC device, suppressing the pump light by 110 dB. Locking is performed every 10 s during which the experiment is paused and continuous wave light is sent to the filter cavities via a fiber-based MEMS optical switch. Each locking procedure takes approximately 1.5 s. The filtered signal from the device containing the optomechanically scattered photons is detected on two superconducting nanowire single-photon detectors (SNSPDs) with dark count rate ∼30 Hz. The electronic signal from the SNSPDs is amplified and recorded by a time-tagging module (TTM).

We calibrate the efficiencies throughout our optical setup, such as lensed fiber to device waveguide coupling ($\eta_{\rm fc} = 0.5$), the coupling between the waveguide and the optical cavity ($\kappa_{\rm e}/\kappa = 0.45$), optical losses in the detection filter setup ($\eta_{\rm filters} = 0.4$) and the efficiencies of the two SNSPDs ($\eta_{\rm SPD1} = 0.61$, $\eta_{\rm SPD1} = 0.55$). The fiber coupling efficiency is measured by measuring the input and output power of the fiber circulator in front of the device. This results in a total detection path efficiency of $\eta = 0.05$.

Figure S2b shows the modified experimental setup of Hong-Ou-Mandel interference measurements. After the optical filters the light is sent through a Mach-Zehnder interferometer with a delay line of 1.43 km length in one

arm. Single-photon detectors are placed at the two outputs of the interferometer. To maximize the interference visibility, the polarization of the two interferometer arms have to be aligned. For polarization alignment, we place two optical switches at one of the interferometer outputs, which can be used to send the light to an optical power meter (PM) and optionally through a polarizing beam combiner (PBC). This PBC features two polarization maintaining fibers at the input which can be used as a reference for polarization alignment. Furthermore, optical attenuators (OA) are used to balance the optical power in both arms. In the following, we describe the detailed procedure for polarization alignment.

- **Co-polarized interferometer arms:** We first minimize the transmission of the long interferometer arm via the OA and send the output light of the interferometer through the PBC. We use the polarization controller (PC) in the short arm to minimize the signal on the PM. We fully attenuate the transmission through the short arm and fully unattenuate the transmission through the long arm. We use the PC in the long arm to again minimize the signal on the PM. We send the output light from the interferometer directly to the PM without going through the PBC by switching the optical switch and measure the output power P_{long} of the long arm. To balance the optical power in the two arms, we fully attenuate the transmission of the long arm again and unattenuate the transmission of the short arm until the measured power $P_{\mathrm{short}} = P_{\mathrm{long}}$. Afterwards, we fully unattenuate the transmission of the long arm. Light passing through the two arms has the same polarization at the second 50:50 BS (co-polarized).

- **Cross-polarized interferometer arms:** The procedure to align the two inteferometer arms to have orthogonal polarization at the second 50:50 BS (cross-polarized) is identical to the alignment procedure for co-polarized measurement except that in between the polarization alignment steps of the two arms we manually change the input fiber of the PBC used as polarization reference. As the two input fibers of the PBC have orthogonal polarizations, after this procedure the two interferometer arms are cross-polarized.

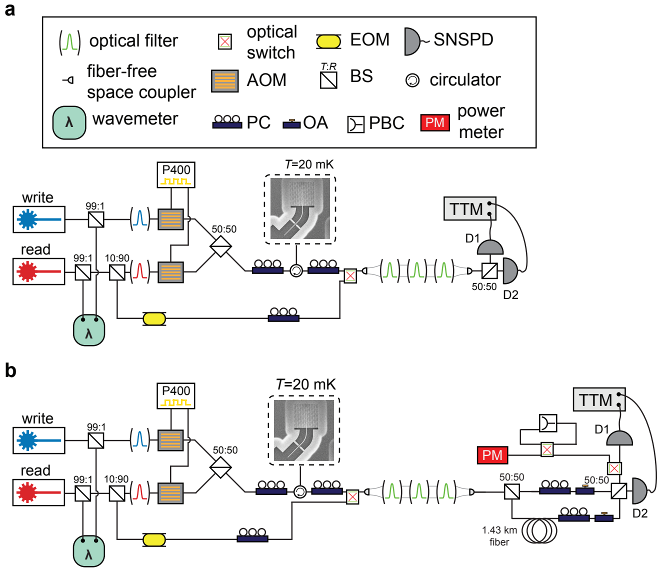

FIG. S2. **Optical setup.** **a** Detailed scheme of the optical setup used for pulsed optomechanical measurements. BS, beam splitter with transmission (reflection) coefficient T (R); AOM, acousto-optic modulator; EOM, electro-optic modulator; TTM, time-tagging module; PC, polarization controller; OA, optical attenuator; PM, optical power meter; PBC, polarizing beam combiner. **b** Modified scheme for HOM measurements.

C. Extended device characterization

Mechanical linewidth measurement

Optomechanically induced transparency (OMIT), resulting from the quantum destructive interference between anti-Stokes scattered field and a weak intracavity pump field [6], can be used to measure the mechanical spectrum as well as mechanical decay rate (Γ_{m}). The induced transparency window (or OMIT linewidth) scales linearly with intracavity photon number n_c. When extrapolating to zero photon number, we obtain $\Gamma_{\mathrm{m}}/2\pi = 119.7\,\mathrm{kHz}$, which matches well with further characterization of the mechanical life time (see below).

Mechanical lifetime measurement

We measure the lifetime of the phonons in the mechanical mode of interest by creating a large coherent phonon population and monitoring its decay over time. Figure S3a shows the pulse sequence used for measuring the lifetime of the mechanical mode. First, a laser pulse (frequency ω_{l}) of duration 40 ns detuned from the optical cavity resonance at ω_{c} by $\Delta/2\pi = (\omega_{\mathrm{l}} - \omega_{\mathrm{c}})/2\pi = 11.5\,\mathrm{GHz}$ and modulated by an electro-optic phase modulator at the mechanical frequency ($\omega_{\mathrm{mod}} = \omega_{\mathrm{m}}$) is sent to the device. The beating between the sideband and carrier frequency of the optical field coherently drives the mechanical mode creating a large phonon population. After a delay time T_{delay}, we read out the phonon population by a second 40 ns long laser pulse detuned on the red optomechanical sideband of the optical cavity. We detect optomechanically scattered photons from the readout pulse on the SNSPDs and vary the delay time be-

tween the two pulses to measure the decay of the coherent phonon population over time. Figure S3b shows the normalized click rate measured on the SNSPDs as a function of T_{delay}. From an exponential fit to the experimental data, we extract the phonon lifetime $\tau = 1.0\,\mu\mathrm{s}$. Figure S3c shows the lifetime measurement of another device with a much longer phonon lifetime of $\tau = 9.0\,\mathrm{ms}$. The significantly increased lifetime of the device in Fig. S3c is achieved by adding an air gap at the end of the device as shown in the inset to reduce radiation loss through the connection to the substrate. For the experiments in the main text, we picked the device in Figure S3b in order to obtain a higher repetition rate.

Higher optical power in general will decrease the mechanical quality factor of the quasi-2D OMC structure. This effect has been studied in earlier works [4], where the mechanical linewidth ($\gamma/(2\pi)$) increased from around 100 kHz to 160 kHz when the intracavity photon number increased from 10^3 to 10^4. Since we only use relatively small intracavity photon numbers in our experiments ($n_{\mathrm{c}} \lesssim 2 \times 10^3$), we expect the modification of the mechanical quality factor in our experiments to be small. Furthermore, for each repetition cycle, $T_{\mathrm{rep}} \gg \tau$, i.e. we wait long enough so the light-induced heat is fully dissipated and the device is fully thermalized to the cold environment (20 mK) before the start of the next repetition cycle. Therefore, a slightly lower mechanical quality factor for higher optical power inside the cavity does not significantly affect our experiments.

Cavity resoanance shift measurement

Importantly, high-power optical laser pulses can lead to shifts in the optical resonance frequency of the OMC device either due to the photothermal effect or free carrier dispersion [7, 8]. A shift in the optical cavity resonance by $\delta\omega_{\mathrm{c}}$ can modify the measured value of Stokes and anti-Stokes scattering probabilities and thus impact the calculated thermal phonon occupancy. We correct the values of anti-Stokes scattering probabilities for this shift by replacing $\Delta \to \Delta - \delta\omega_{\mathrm{c}}$ in main text Eq. (4).

To calibrate the shift $\delta\omega_{\mathrm{c}}$ in our device, we use a pump-probe measurement of the optical cavity resonance frequency. The pulse sequence used for this experiment is shown in Fig. S4a. We send optical pump pulses detuned to the red optomechanical sideband of the optical cavity with pulse length 40 ns and, simultaneously, a second strongly attenuated probe pulse with pulse length 5 µs to the device. This pulse sequence is repeated with a repetition period of $T_{\mathrm{rep}} = 10\,\mu\mathrm{s}$. We lock the free-space optical filters to the wavelength of the probe pulse to remove the strong pump light and measure the reflected probe signal from the optical cavity on the SNSPDs.

By varying the wavelength of the probe pulse we can measure the optical cavity resonance frequency as a

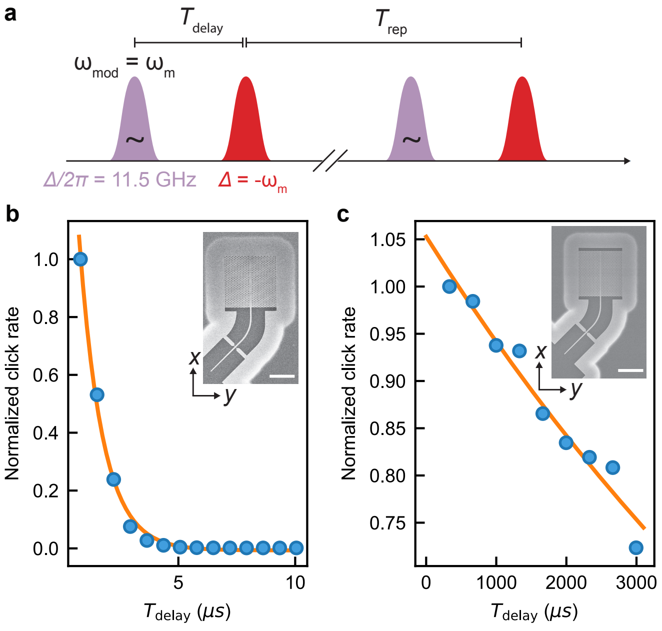

FIG. S3. **Measurement of phonon lifetime. a** Pulse sequence used to measure the phonon lifetime. A first pulse detuned from the optical cavity resonance by $\Delta/2\pi = 11.5\,\mathrm{GHz}$ is modulated by an electro-optical phase modulator at the mechanical frequency ω_{m}. After a variable delay time of T_{delay}, a readout pulse detuned to the red optomechanical sideband reads out the phonon population in the mechanical mode. The pulse sequence is repeated with repetition period T_{rep}. **b** Click rate measured on SNSPDs during the readout pulse normalized to the first data point as a function of delay time T_{delay}. Solid orange line is a fit to an exponential decay yielding a lifetime of phonons in the mechanical mode of $\tau = 1.0\,\mu\mathrm{s}$. **c** Normalised click rate as a function of delay time T_{delay} from another device with a much longer lifetime. The exponential fit gives a lifetime $\tau = (9.0 \pm 0.7)\,\mathrm{ms}$. Insets in **b** and **c** show SEM images of the respective devices with scale bars corresponding to 10 µm.

function of time (see Fig. S4b). When the pump pulse arrives after approximately 0.1 µs, a blue shift of the cavity resonance is observed. While the exact mechanism of this blue shift warrants further investigation in the future, it is likely due to free carrier dispersion effects routinely observed in silicon photonic crystal devices [7]. We extract the value of the cavity resonance shift $\delta\omega_{\mathrm{c}}$ by binning the data in Fig. S4b in 5 ns bins and fitting Lorentzian lineshapes to extract the time-resolved cavity resonance frequency as shown in Fig. S4c. We vary the power of the pump pulse over the typical range used in the measurements presented in the main text (see Fig. S4d). We use the data in Fig. S4d to correct the measured values of the Stokes and anti-Stokes scattering probability as well as the obtained value of the thermal phonon occupancy in Eq. (6) in main text.

Aside from cavity resonance shift, changes of the op-

tical quality factor in response to different powers has been studied in [9], where different non-linear effects (such as two-photon absorption and free carrier absorption) due to the high electromagnetic energy stored in the OMC structure have been incorporated. When the optical power loaded to the cavity is increased to hundreds of µW, it will eventually lead to a broadening of the optical linewidth. However, even for the highest power loaded into the cavity used in our experiment (∼1.6 fW), the change in optical quality factor is negligible according to the simulation in [9], which is consistent with our observations in the experiment. Moreover, considering we are deep in the sideband-resolved regime (mechanical frequency $\omega_m/2\pi \sim 10$ GHz and optical linewidth $\kappa/2\pi \sim 2.4$ GHz), the resulting change from optical linewidth in scattering probabilities is even more negligible.

Measurement of the thermal phonon occupancy due to delayed heating from the write pulse

For measuring quantum correlations between phonons and photons as well as measurements of the photon autocorrelation function and Hong-Ou-Mandel interference as presented in the main text, we use a pulse sequence consisting of a blue-detuned write pulse followed by a red-detuned readout pulse. We calibrate the additional thermal phonon population induced by the write pulse by using a pulse sequence consisting of two pulses as shown in Fig. S5a: the first red-detuned pulse creates thermal phonons. Note that we use a red pulse for this, whereas the write pulse in the experiments in the main text is blue-detuned. The thermal phonon occupancy induced by a blue- or red-detuned pulse is expected to be the same considering the same amount of intracavity photon number for blue- and red-detuned pulses at the same power. Hence, we choose to use a red-detuned pulse to avoid additional phonon occupancy by optomechanical driving through the two-mode squeezing interaction of a blue-detuned pulse. The second pulse is either blue- or red-detuned and is used to measure the phonon occupancy created by the first pulse through sideband asymmetry. For small optomechanical scattering probabilities such that $p_{aS} \approx p_S$ in Eqs. (3) and (4) in main text, it follows from Eqs. (1) and (2) in main text that the thermal phonon occupancy n during the second pulse can be calculated from the asymmetry in count rates during the second pulse when using either a blue-detuned (C_S) or red-detuned pulse (C_{aS}) as

$$n_{th} = \frac{1}{C_S/C_{aS} - 1}. \tag{S2}$$

Figure S5b shows the measured click probability on the SNSPDs during the second pulse with the laser detuned on the blue or red optomechanical sideband as a function of optical power or equivalently Stokes scattering probability of the first pulse. From Eq. (S2), we calculate the corresponding thermal phonon occupancy during the second pulse as shown in Fig. S5c. The value of n_{th} obtained in this way contains both contribution from thermal phonons created due to the first (n_{p1}) and second pulse (n_{p2}). The optical power of the second pulse is fixed to 100 nW. Therefore, we can isolate the contribution of the first pulse by subtracting the contribution of the second pulse which is known from the calibration of the thermal phonon occupancy shown

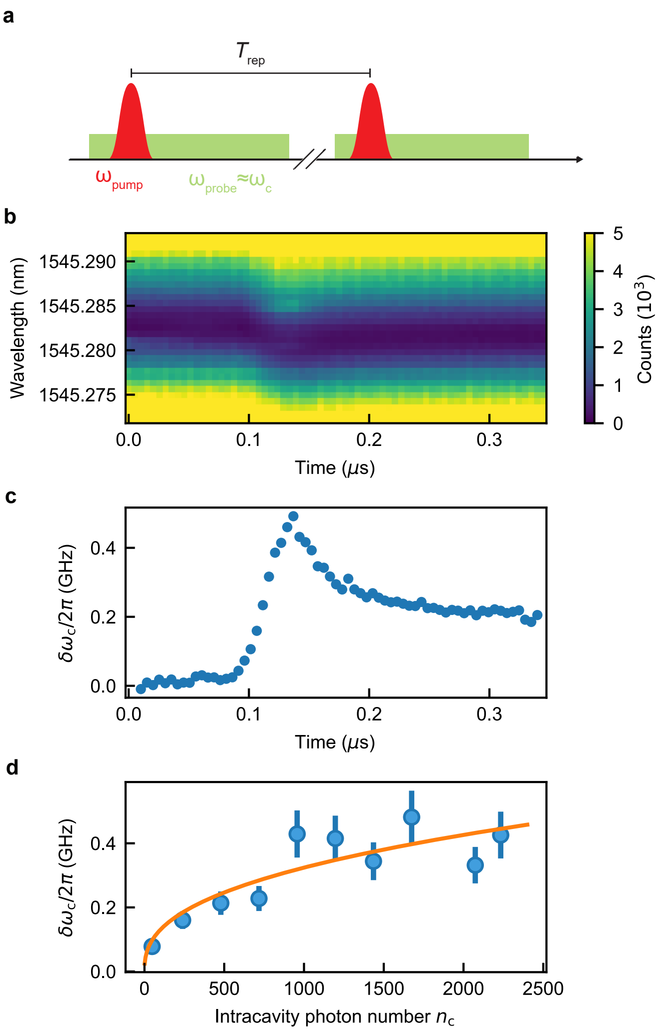

FIG. S4. **Calibration of the optical cavity shift. a** Pulse sequence used for optical cavity shift calibration measurement. **b** Integrated counts on SNSPDs as a function of time while varying the wavelength of the probe laser at input laser power of 1400 nW. **c** Time-resolved shift of the optical cavity resonance frequency $\delta\omega_c$ extracted from the data in **b**. **d** Maximum shift of the optical cavity resonance frequency $\delta\omega_c$ measured at various intracavity photon number n_c. Solid orange line is a fit to a power law $f(x) = ax^A$ with coefficients $a = 0.025$, $A = 0.39$.

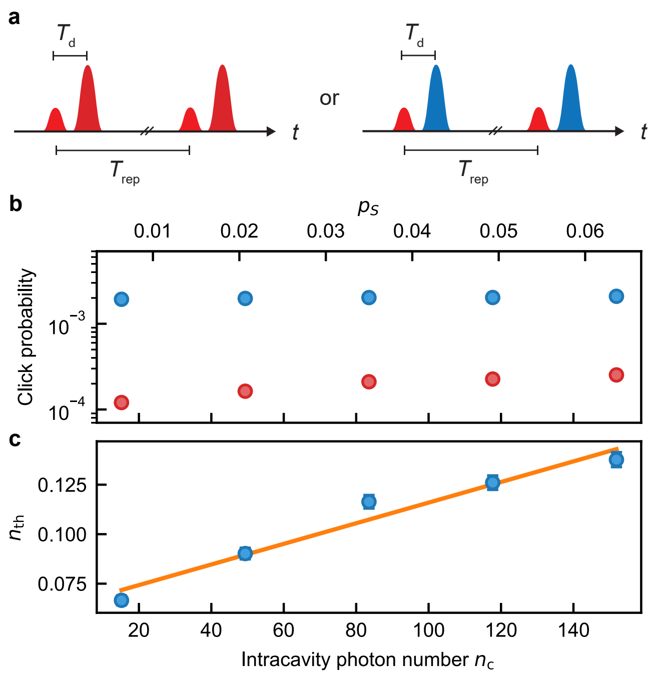

FIG. S5. **Calibration of the added thermal phonons from write pulse.** **a** Pulse sequence for sideband asymmetry measurement with a prepulse to calibrate the thermal phonon occupancy added by the prepulse. The delay between the two pulses is $T_d = 150\,\mathrm{ns}$ as is used as the delay between the write and read pulse in the cross-correlation and HBT measurements presented in the main text. The prepulse is detuned on the red optomechanical sideband ($\Delta = -\omega_m$). We repeat the experiment with the second pulse for sideband asymmetry either blue- ($\Delta = \omega_m$) or red-detuned ($\Delta = -\omega_m$). The power of the second pulse is fixed to an intracavity photon number of $n_c = 300$. **b** Measured click probability during the second pulse when the laser is detuned to the blue or red optomechanical sideband and **c** calculated thermal phonon occupancy n_{th} as a function of intracavity photon number (Stokes scattering probability) of the prepulse on the bottom (top) axis. The solid orange line is a linear fit to the data.

in the main text to be $n_{p2} = 0.047$. Both the cross-correlation as well as the HBT measurements shown in the main text use a write pulse power of intracavity photon number $n_c = 30$ (Stokes scattering probability $p_S = 1.3\%$). For this write pulse power, we obtain $n_{write} = n_{p1} = n_{th}(n_c = 30) - n_{p2} = 0.039$.

D. Extended discussion of improved thermalization of 2D optomechanical crystal cavities

A phenomenological model of optical absorption heating and the generation of thermal noise in silicon OMCs has been established in earlier work [10, 11]: due to the existence of dangling bonds at the silicon/silicon oxide interface, discrete interface defect states below and above the silicon electronic midgap can be formed due to unreacted Si dangling bonds or Si dangling bonds interacting with oxygen atoms [12–14]. It is believed that upon absorption of telecom photons through the interface states, a high-frequency (THz) thermal phonon bath is generated, which will go through three-phonon scattering processes involving the mechanical mode of interest and the local thermal bath, where phonons of GHz frequency will be generated through the interaction between two high-frequency phonon modes [15, 16]. The general design strategy behind the 2D OMC structure is to make use of the higher density of phononic states resulting from a large contact area with the substrate that can carry away heat before the GHz mechanical mode of interest is populated [2]. In [2], the difference in thermal conductance between 1D nanobeam and 2D OMC structures has been simulated, and a factor of 42 enhancement was expected in the thermal conductance coefficient for the quasi-2D OMC, which was believed to be the main reason for the improvement in thermal performance.

Based on the calibration of thermal occupancy as well as the optomechanical sideband thermometry measurement presented in the main text, it is evident that the 2D OMC devices used in this work exhibit reduced thermal noise at the same intracavity photon numbers compared to 1-dimensional nanobeam OMCs. While we observe a factor of three reduction in thermal noise in the devices in our work, we compare the thermal performance of different 2D OMC structures here in more detail. For direct comparison, according to the data in Figure 4(c) in [4], with scattering probability of approx. 5% and photon-phonon pair generation rate of 5 kHz, the estimated phonon occupation is between 0.02 to 0.03. For our Figure 2(a) in the main text, with scattering probability of 5%, and a photon-phonon pair generation rate of 5 kHz (since our repetition rate is 100 kHz), the measured thermal phonon occupancy is 0.029, which is very comparable to [4]. We would like to note that there can still be some variation in thermal performance between individual devices due to fabrication imperfections.

For the side-coupled snowflake + Cshape structure investigated in [17] with a repetition rate of 250 Hz, at 10% transduction efficiency, the phonon occupancy is around 0.045, while at 90% transduction efficiency the phonon occupancy is 0.25. For our end-coupled snowflake + Cshape structure, with a repetition rate of 100 kHz, at 10% transduction efficiency, the phonon occupation is approx. 0.041; at 60% transduction efficiency, the phonon occupation is 0.3. We can see that at high power and high transduction efficiency, the side-coupled structure performs better than our device used in the experiments (despite the different repetition rates used here). This is mainly due to the side-coupled geometry that is used in [17], which mechanically detaches the optical cavity from the coupling waveguide which could act as a source of thermal noise [17]. We note that alternative

approaches to achieving such evanescent coupling to 2D OMC devices exist, for example by using pick-and-place techniques to transfer photonic coupling waveguide structures on top of the 2D OMC [18].

E. Optomechanical single-photon generation protocol

As a first step, we generate single phonons in the mechanical mode through a pulsed optomechanical interaction, in combination with single-photon detection [19]. A laser pulse detuned on the blue optomechanical sideband of the optical cavity resonance induces a two-mode squeezing interaction between the optical and mechanical mode with interaction Hamiltonian $H_{\mathrm{tms}} = \sqrt{n_{\mathrm{c}}} g_0 (a^\dagger b^\dagger + ab)$, where a (b) are the annihilation operators of the optical (mechanical) mode and n_{c} is the intracavity photon number. This corresponds to a Stokes scattering process, in which the optomechanical interaction converts a photon from the blue-detuned pump into a photon at the optical cavity resonance and a phonon in the mechanical mode. After the interaction, the joint system is in the state

$$|\psi\rangle = \sqrt{1 - p_{\mathrm{S}}} \sum_{n=0}^{\infty} \sqrt{p_{\mathrm{S}}}^{\,n} \, |nn\rangle \, , \tag{S3}$$

where p_{S} is the optomechanical scattering probability for the Stokes process. The light coming back from the device is filtered by a series of optical filters to remove the strong blue-detuned pump and detect single optomechanically scattered photons on superconducting nanowire single-photon detectors (SNSPD). The detection of a single-photon projects the mechanical mode into the state

$$|\psi_{\mathrm{m}}\rangle = \sqrt{\frac{1 - p_{\mathrm{S}}}{p_{\mathrm{S}}}} \sum_{n=1}^{\infty} \sqrt{p_{\mathrm{S}}}^{\,n} \, |n\rangle \, , \tag{S4}$$

which for small scattering probability $p_{\mathrm{S}} \ll 1$ is close to a single phonon Fock state $|\psi_{\mathrm{m}}\rangle \approx |1\rangle$. Thus, the emitted photon from the Stokes process heralds the creation of the single phonon state. After the phonon creation, a second laser pulse detuned to the red optomechanical sideband transfers the phonon state onto the optical mode through a beam-splitter interaction with interaction Hamiltonian $H_{\mathrm{bs}} = \sqrt{n_{\mathrm{c}}} g_0 (a^\dagger b + a b^\dagger)$. This corresponds to an anti-Stokes scattering process in which a photon from the red-detuned pump and a phonon in the mechanical mode scatter into a photon at the optical cavity resonance. This mechanics-to-optics conversion process allows us to generate single photons after filtering out the strong red pump pulse.

F. Hanbury Brown-Twiss measurements

Definition and measurement of the second-order intensity autocorrelation function $g^{(2)}(\tau)$

In the Hanbury Brown-Twiss (HBT) measurement in the main text, we measure the intensity autocorrelation function of the optical anti-Stokes field generated by the optomechanical beam splitter interaction during the red-detuned readout pulse. The evaluation of the autocorrelation function is conditioned on the detection of a Stokes-scattered photon during the blue-detuned write pulse, which heralds the creation of a single phonon in the mechanical mode and thus represents a third-order correlation function. After heralding, the conditional second-order autocorrelation function used to characterize the purity of the generated single photons from the optomechanical anti-Stokes scattering process is defined as [20]

$$g^{(2)}(\tau) = \frac{\langle a^\dagger(0) a^\dagger(\tau) a(\tau) a(0)\rangle}{\langle a^\dagger(\tau) a(\tau)\rangle \langle a^\dagger(0) a(0)\rangle}, \tag{S5}$$

where τ is the time delay between a coincidence detection on detector 1 and detector 2 in the HBT setup.

In the regime of low photon detection probability [21] and for $\tau = 0$, the theoretical definition of the photon autocorrelation function in Eq. (S5) can be connected to the probability of detecting coincidence clicks on the two outputs of a beam splitter in a HBT configuration as discussed in the main text. In this case, Eq. (S5) reduces to the cross-correlation between the two detectors [20]

$$g^{(2)}(0) = \frac{P(\mathrm{D}_1 \cap \mathrm{D}_2)}{P(\mathrm{D}_1) P(\mathrm{D}_2)}, \tag{S6}$$

where $P(\mathrm{X})$ describes the probability of detection event X. Event $\mathrm{D}_1 \cap \mathrm{D}_2$ represents the coincidence detection on both detectors heralded on a Stokes photon click, and event $\mathrm{D}_1(\mathrm{D}_2)$ represents a single click on detector $\mathrm{D}_1(\mathrm{D}_2)$ heralded on a Stokes photon click.

To illustrate the anti-bunching behavior of optomechanically generated single photons, we also calculate the value of the conditional autocorrelation function $g^{(2)}(\tau)$ for detection events in different repetitions of the experiment where $\tau = T_{\mathrm{rep}} \Delta n$ with the repetition time of the experiment $T_{\mathrm{rep}} = 10\,\mu\mathrm{s}$. The integer offset Δn corresponds to the number of repetitions of the experiment between two clicks on detectors D1 and D2 for which heralding was successful. Hence, Δn represents the stochastically varying time delay between two such detection events. Such detection events are fully uncorrelated and are thus expected to yield $g^{(2)}(\Delta n \neq 0) = 1$ as shown in the main text.

The error bars of the conditional autocorrelation function are calculated from the single-photon counting statistics by evaluating the exact binomial confidence interval using the Clopper-Pearson method to account for

the asymmetry of the binomial distribution due to the low number of successful detection events.

Criteria for non-classicality and single-photon generation

The autocorrelation function in Eq. (S5) characterizes the statistical properties of the anti-Stokes scattered light field described by the annihilation operator a. The value of the autocorrelation function at $\tau = 0$ serves as a metric to proof the non-classical nature of an optical state: for classical light fields, the autocorrelation function is bound to $g^{(2)}(0) \geq 1$ and hence a value of $g^{(2)}(0) < 1$ indicates a non-classical state of light [22]. An even more stringent bound can be derived to proof the single-photon nature of an optical state: for a photon number state $|n\rangle$, Eq. (S5) yields

$$g^{(2)}(0) = \frac{\langle n(n-1)\rangle}{\langle n\rangle^2} = \frac{n-1}{n}. \tag{S7}$$

Equation (S7) shows that $g^{(2)}(0) = 0$ for an ideal single-photon Fock state $|n = 1\rangle$, whereas $g^{(2)}(0) \geq 0.5$ for any higher Fock state $n \geq 2$. Therefore, $g^{(2)}(0) < 0.5$ has become a standard criterion to demonstrate the single-photon nature of an optical state [23]. Since the anti-Stokes photons are created by mechanics-to-optics conversion through an optomechanical beam splitter interaction, the autocorrelation function in Eq. (S5) also directly measures the second-order intensity correlation of the mechanical mode. Therefore, demonstrations of $g^{(2)}(0) < 1$ and $g^{(2)}(0) < 0.5$ imply non-classical and single-phonon states in the mechanical mode, respectively.

Impact of dark counts

The impact of dark counts on the measured $g^{(2)}(0)$ value is evaluated by choosing a time window of same length but 80 ns after the optomechanical signal. The rate of the dark counts is in general less than 2% of both the Stokes scattered photon count rate and the anti-Stokes scattered photon count rate, which results in 0.78% of coincidences.

Impact of SNSPD dead time

If the pulse delay between the write and read pulse is shorter than the dead time of the superconducting nanowire single-photon detectors (SNSPD), this can lead to incorrect values of the observed cross- and auto-correlation functions. In particular, in Hanbury Brown-Twiss measurements this would lead to a measured value of the auto-correlation function $g^{(2)}(0)$ that is lower than

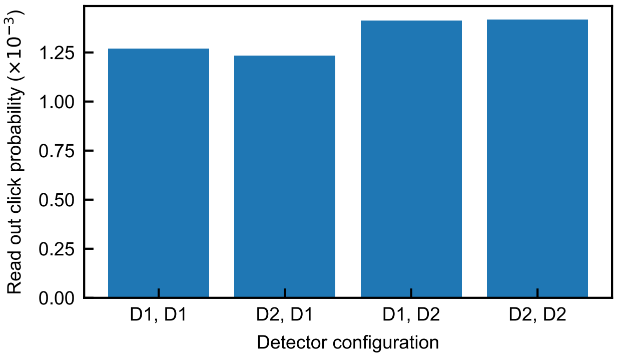

FIG. S6. **Impact of SNSPD dead time on HBT measurements.** Click probability on SNSPDs during the read out pulse after heralding for different detector configurations. Detector configurations are labeled as D_i, D_j, where D_i (D_j) indicates the detector used for heralding (readout).

the true value. To rule out the impact of the SNSPD dead time, we consider the probability to detect a photon during the read out pulse on one detector after heralding for different detector configurations (see Fig. S6). The different detector configurations are labeled as D_i, D_j, where D_i (D_j) indicates the detector used for heralding (readout). The probability to detect a photon on D_1 does not depend significantly on whether D_1 or D_2 was used for heralding. This implies that after the heralding photon is detected on D_1, the detector has already recovered to its full detection efficiency before the read out photon arrives. The same observation can be made for the remaining two detector configurations in which the readout photon is detected on D_2. The slightly lower detection probability between the configurations where the read out photon is detected on D_1 compared to the configurations where is detected on D_2 can be explained by the slightly higher detection efficiency $\eta_1 = 0.55$ compared to $\eta_2 = 0.61$.

G. Hong-Ou-Mandel interference

Calibration of interference visibility using weak coherent light

To calibrate for the imperfect power balance of the two arms in the Hong-Ou-Mandel (HOM) setup, we measure the HOM interference of weak coherent light in the co- and cross-polarized cases. Weak coherent light pulses at optical cavity resonance are generated and sent through the same HOM setup for interference. In S7a, we can see the dip in the co-polarized case. It represents a visibility of $V = 0.464$. The deviation from 0.5 visibility is mainly attributed to the power imbalance of the two arms. This ratio is used to correct the measured HOM

visibility of optomechanically generated single photons (see main text). The smaller dips on both sides result from the relatively long coherence time of the coherent laser light used in this measurement.

In the HOM measurement of single photons (see main text), if we do not perform any heralding on the Stokes photons from the blue-detuned pulse and directly analyze the HOM interference of anti-Stokes photons from the red-detuned pulse, this corresponds to the HOM interference of thermal light. In S7b, we can see there is a peak in the cross-polarized case. The ratio between the peak and the side bars is 1.35, which matches well with the Qutip simulation considering the amount of thermal component introduced in our pulse scheme (see QuTiP Simulations below).

Modeling of temporal wavepacket shape

In the main text we measure the HOM interference between an optomechanically generated single photon and a thermal photon state as a function of timing offset Δt of the red-detuned read out pulses. Here, we introduce a phenomenological model to describe the dependence of the HOM dip on Δt. The depth of the HOM dip depends on the overlap of the two wavepackets of the photons impinging on the beam splitter, which is given by a convolution of the wavepacket envelops $P_1(t)$ and $P_2(t)$. We model the normalized number of coincidences $g_{\mathrm{HOM}}(\Delta t)$ by

$$g_{\mathrm{HOM}}(\Delta t) = A\left(1 - B\int_{-\infty}^{\infty} P_1(t)P_2(t-\Delta t)\right), \quad \text{(S8)}$$

where A and B are fitting parameters to model the relative depth of the HOM dip. The pulse shape of the photon wave packet follows the pulse shape of the red readout pulse, which is created by applying a square gating voltage pulse of length T_{read} to an acousto-optic modulator with $f_{\mathrm{AOM}} = 110\,\mathrm{MHz}$ operating frequency. The relatively slow cutoff time constant of the AOM ($\tau_{\mathrm{AOM}} = 1/f_{\mathrm{AOM}} \approx 9.1\,\mathrm{ns}$) leads to significant broadening of the pulse length compared to the nominal values of 40 ns or 100 ns used in the experiment. Thus, we model the pulse shape by convoluting the square pulse with a Lorentzian with width τ_{AOM} in the time domain.

As mentioned in the main text, for increasing pulse offset Δt we measure a normalized number of coincidences larger than unity. The number of coincidences is normalized to the value measured on the satellite peaks with uncorrelated coincidences where $\Delta n \neq 0$. When, the timing offset increases, the overlap of the two readout pulses is reduced. The parts of the two pulses that are not overlapping in time cannot interfere. Therefore, the coincidence events detected in the non-overlapping part of the pulses measure the photon statistics of the incoming photon state in an HBT-type experiment. As the measured coincidence number is measured within the whole pulse window of both readout pulses, all of these coincidence events overlap. When the two pulses are completely separated in time, this measurement measures the overlapping coincidences from two simultaneous HBT experiments on a thermal state and an optomechanically generated single photon giving rise to a normalized coincidence number larger than 1 due to the bunched photon statistics of the thermal state.

H. QuTiP simulations

We use the Python package QuTiP to simulate the expected values of the cross-correlation, auto-correlation functions [24, 25], and the results of HOM interference measurements.

Simulation procedure

We keep track of the full evolution of the density matrix of the mechanical and optical mode of the system throughout a pulse sequence consisting of first a blue-detuned and secondly a red-detuned pulse as used for the measurements in the main text with respective optomechanical scattering probabilities p_{S} and p_{aS} for the induced Stokes and anti-Stokes scattering processes. More details of the simulation procedure can be found in [26]. We include the following imperfections and sources of loss in our simulation:

1. We include the thermal occupancy of the mechanical mode both originating from the write and readout pulse. We include the thermal occupancy due to the write pulse by initiating the mechanical mode in a thermal state with thermal occupancy n_{write} in the beginning of the simulation.

2. We include the additional thermal occupancy added through the readout pulse by coupling the system to a thermal bath with phonon occupancy n_{read}. The coupling is modeled by a two-mode squeezing interaction with squeezing angle $\sqrt{n_{\mathrm{read}}}$ between the mechanical mode and an auxiliary vacuum mode in the environment and then tracing out the auxiliary mode. The coupling is assumed to occur right before the readout pulse.

3. In the time period between the write and read pulses, phonons in the mechanical mode may decay due to the limited lifetime of the mechanical mode $\tau = 1.0\,\mu\mathrm{s}$. We account for this by applying an amplitude damping channel with loss probability $p_{\mathrm{loss}} = e^{-\tau/T_{\mathrm{delay}}}$, where T_{delay} is the delay between the write and read pulses.

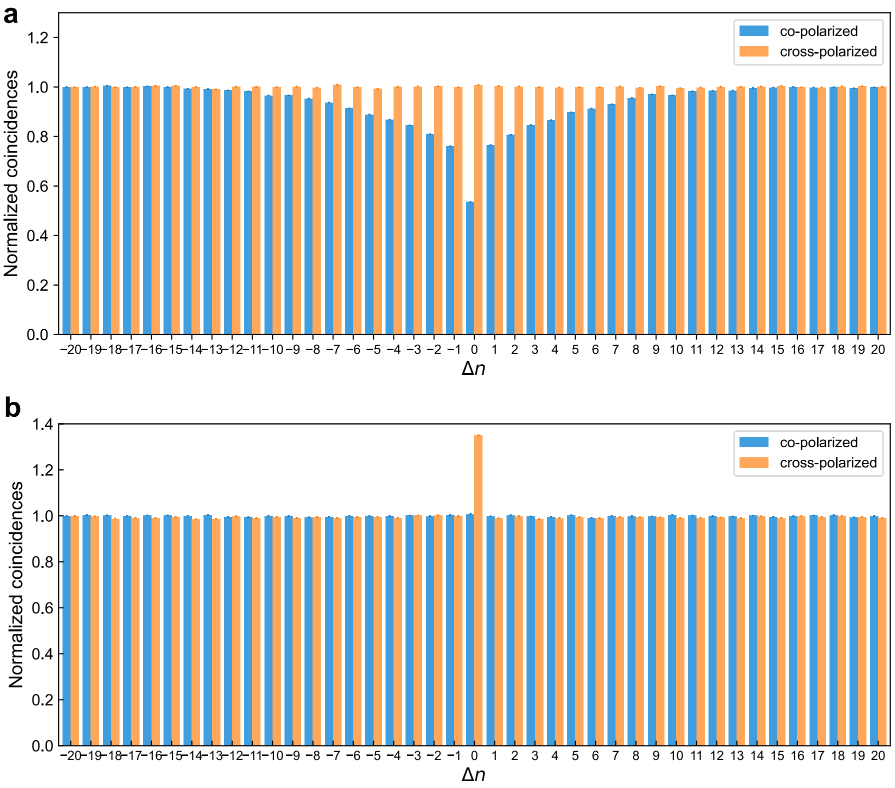

FIG. S7. **HOM interference weak coherent light and thermal light.** HOM interference measurement of weak coherent light (**a**) and thermal light (**b**). In the weak coherent light measurement, visibility $V = 0.464$, the deviation from 0.5 visibility is mainly attributed to the power imbalance of the two arms. In the thermal light analysis, the ratio between the peak and the side bars is 1.35 in the cross-polarized case, which matches well with the Qutip simulation considering the amount of thermal component introduced in our pulse scheme (see QuTiP Simulations below).

4. After the mechanical state is transferred to the optical state by optomechanical readout, we apply an amplitude damping channel to account for optical losses on the detection path with loss probability $p_{\mathrm{loss}} = 1 - \eta$, where η is the efficiency of the detection path.

We denote the optical output state after following this simulation procedure as $\rho_{\mathrm{om,out}}(p_{\mathrm{S}}, p_{\mathrm{aS}}, n_{\mathrm{write}}, n_{\mathrm{read}})$.

Simulation of cross- and auto-correlation functions

We evaluate the resulting cross-correlation between detection events during the write and readout pulse $g^{(2)}_{\mathrm{S,aS}}$ and autocorrelation function of the readout optical field $g^{(2)}(0)$. Figure S8a shows the simulated cross-correlation function as a function of total thermal phonon occupancy during the readout pulse. The total thermal phonon occupancy is given by

$$n_{\mathrm{th}} = n_{\mathrm{write}} + n_{\mathrm{read}}, \tag{S9}$$

where n_{write} and n_{read} are the thermal phonon populations induced due to heating during the write pulse and read pulse. To compare the simulations to the measurements we fix the Stokes scattering probability during the write pulse to $p_{\mathrm{S}} = 1.3\%$ as used in the cross-correlation and HBT measurements. As calibrated above, the write pulse creates a thermal phonon population of $n_{\mathrm{write}} = 0.039$. We vary the value of anti-Stokes scattering probability during the red-detuned readout pulse and use the calibrated thermal phonon occupancy shown in the main text for n_{read}.

The maximum observable value of the cross-correlation function is limited by higher-order phonon creation events from the two-mode squeezing interaction during the write pulse limiting $g^{(2)}_{\mathrm{S,aS}}$ as

$$g^{(2)}_{\mathrm{S,aS}} = 1 + \frac{1}{p_{\mathrm{S}}}, \tag{S10}$$

where p_{S} is the Stokes scattering probability. In accordance with previous works [19], we find that the presence of an additional thermal phonon population n_{th} reduces the observable cross-correlation as

$$g^{(2)}_{\mathrm{S,aS}} = 1 + \frac{e^{-T_{\mathrm{delay}}/\tau}}{p_{\mathrm{s}} + n_{\mathrm{th}}}. \tag{S11}$$

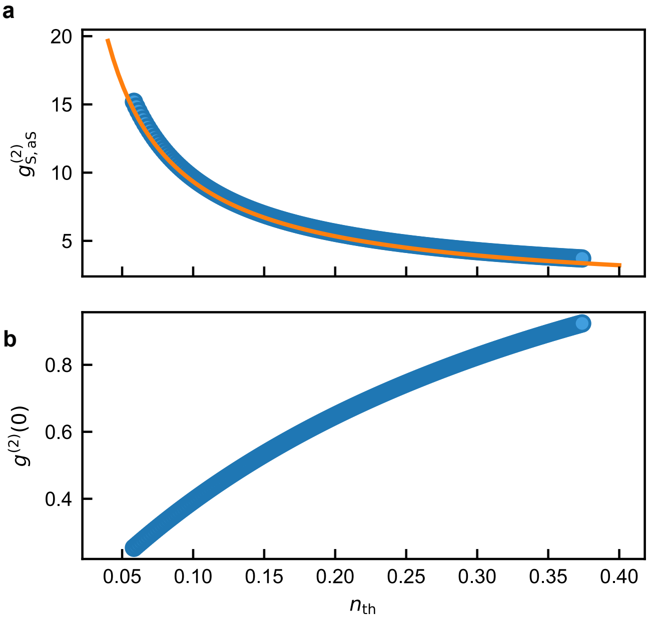

FIG. S8. **Qutip simulations of cross- and auto-correlation functions.** **a** Simulated cross-correlation function $g^{(2)}_{\mathrm{S,aS}}$ between write and read pulse as a function of total thermal population n_{th}. In the simulation, the Stokes scattering probability is fixed at $p_{\mathrm{S}} = 1.3\%$ and the anti-Stokes scattering probability during the readout pulse p_{aS} is varied. The orange line corresponds to the phenomenological expression in Eq. (S11). **b** Simulated autocorrelation function $g^{(2)}(0)$ of the optical readout field as a function of n_{th}.

The factor $e^{-T_{\mathrm{delay}}/\tau}$ accounts for phonon decay due to the finite phonon lifetime. Equation (S11) is shown as an orange line in Fig. S8 and shows excellent agreement with the simulated values of expected $g^{(2)}_{\mathrm{S,aS}}$.

Figure S8b shows the simulated autocorrelation function $g^{(2)}(0)$ as a function of thermal phonon occupancy n_{th}. We estimate that the measured value of the photon autocorrelation function can be improved to $g^{(2)}(0) = 0.09$ using OMC devices with improved device geometry that reduce the thermal phonon occupancy by a factor of six at equal scattering probabilities [17].

Simulation of HOM interference

To simulate the result of the HOM interference measurements, we define four optical modes corresponding to horizontal (H) and vertical polarizations (V) in the two arms of the Mach-Zehnder interferometer (long: L, short: S) each with Hilbert space size N. The joint density matrix of all modes is defined as $\rho_{\mathrm{in}} = \rho_{\mathrm{L,H}} \otimes \rho_{\mathrm{L,V}} \otimes \rho_{\mathrm{S,H}} \otimes \rho_{\mathrm{S,V}}$. Since our detection scheme is insensitive to the polarization of the detected photons, we mix the two polarization modes in each of the arms by applying a beam splitter interaction and then interfere modes of the same polarization by applying another beam splitter interaction. After the interference, we simulate polarization-insensitive detection of photons in the two output modes 1 and 2 by implementing the following projection operators:

$$P^{(N^2)}_{\neq|0\rangle} = I^{(N)} \otimes I^{(N)} - (|0\rangle\langle 0| \otimes |0\rangle\langle 0|)^{(N)} \quad \text{(S12)}$$

$$P^{(N^4)}_{\neq|0\rangle,1/2} = P^{(N^2)}_{\neq|0\rangle} \otimes I^{(N)} \otimes I^{(N)} \quad \text{(S13)}$$

$$P^{(N^4)}_{\neq|0\rangle,\mathrm{both}} = P^{(N^2)}_{\neq|0\rangle} \otimes P^{(N^2)}_{\neq|0\rangle}, \quad \text{(S14)}$$

where the superscript indicates the dimension of the Hilbert space the operator is acting on. The operator $P^{(N^4)}_{\neq|0\rangle,1/2}$ projects the system into the state after detection of a single click at the detector at output 1 or 2 of the interferometer, whereas $P^{(N^4)}_{\neq|0\rangle,\mathrm{both}}$ projects onto the state after a coincidence detection at both detectors. The corresponding probabilities for single click ($p_{\mathrm{D1,D2}}$) or coincidence detection (p_{c}) are calculated as:

$$p_{\mathrm{D1,D2}} = \mathrm{Tr}\left(P^{(N^4)}_{\neq|0\rangle,1/2}\rho_{\mathrm{HOM,out}}\right) \quad \text{(S15)}$$

$$p_{\mathrm{c}} = \mathrm{Tr}\left(P^{(N^4)}_{\neq|0\rangle,\mathrm{both}}\rho_{\mathrm{HOM,out}}\right), \quad \text{(S16)}$$

where $\rho_{\mathrm{HOM,out}}$ is the joint density matrix of all four output modes. We define the second-order correlation function of detection events on the single-photon detectors as

$$g^{(2)}_{\mathrm{HOM}} = \frac{p_{\mathrm{c}}}{p_{\mathrm{D1}}p_{\mathrm{D2}}}, \quad \text{(S17)}$$

which corresponds to the normalized number of coincidence clicks as presented for the measurement result in the main text.

Figure S9a shows the result of QuTiP simulations using the procedure described above with both mechanical modes initialized in thermal states with varying thermal phonon occupancy n_{th} either in co-polarized or cross-polarized configuration. We observe $g^{(2)}_{\mathrm{HOM}} > 1$ for the cross-polarized measurement configuration irrespective of n_{th}. The slight decrease of $g^{(2)}_{\mathrm{HOM}}$ with increasing n_{th} in the simulations is an artefact of the truncated Hilbert space dimension ($N = 7$) limiting the accuracy of the simulation for large thermal states. In this case, interference of the modes in the two interferometer arms occurs for each polarization independently. As our detection scheme is not sensitive to the photon polarization, this case corresponds to simultaneous HBT measurements on each of the input modes. Detection events of the two HBT measurements overlap giving rise to $g^{(2)}_{\mathrm{HOM}} > 1$ due to the thermal nature of the input state. For co-polarized measurement configuration, the modes in the two arms interfere reducing the probability of coincidence detection events. The result of the QuTiP simulations are in reasonable agreement with the experimental data point

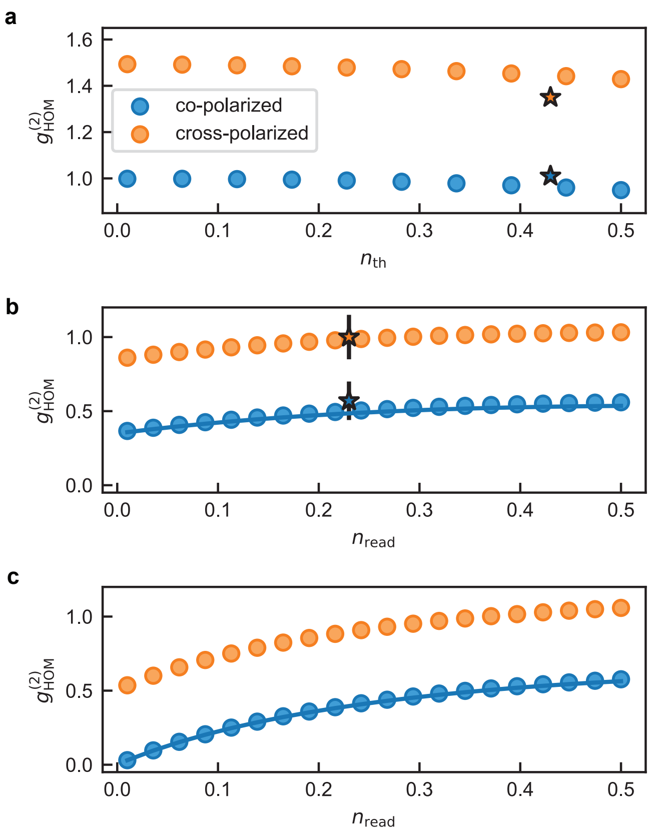

FIG. S9. **Qutip simulations of HOM interference.** **a** Second order correlation function for the HOM measurement $g^{(2)}_{\mathrm{HOM}}$ for thermal states as a function of thermal phonon occupancy n_{th}. **b** Second order correlation function for the HOM measurement $g^{(2)}_{\mathrm{HOM}}$ for optomechanically generated single photons with density matrix $\rho_{\mathrm{om,out}}(p_{\mathrm{S}}, p_{\mathrm{aS}}, n_{\mathrm{write}}, n_{\mathrm{read}})$ as a function of thermal phonon occupancy added during the readout pulse n_{read} with other simulation parameters fixed at the parameters used for the experiment presented in the main text $p_{\mathrm{S}} = 0.1$, $n_{\mathrm{write}} = 0.2$. Stars in **a** and **b** indicate experimental data points from the HOM measurement using two thermal states (see Fig. S7b) and optomechanically generated single photons (see main text). For the measured data point in **a**, the errorbar is smaller than the marker size. **c** Second order correlation function for the HOM measurement $g^{(2)}_{\mathrm{HOM}}$ for optomechanically generated single photons as a function of thermal phonon occupancy added during the readout pulse n_{read} for the ideal case of low Stokes scattering probability $p_{\mathrm{S}} = 0.001$ and no initial thermal phonon occupancy from the write pulse $n_{\mathrm{write}} = 0$. The solid line in **b** and **c** is the analytical approximation in Eq. (S26) based on the auto-correlation function $g^{(2)}(0)$ of the state $\rho_{\mathrm{om,out}}$.

(indicated by a star in Fig. S9a) from the HOM measurement of two thermal states (see Fig. S7)b). The slightly lower value of $g^{(2)}_{\mathrm{HOM}}$ measured in the experiment compared to the numerical simulations may be due to imperfect balancing of the optical power in the two interferometer arms as discussed above.

To model the realistic case of HOM interference of optomechanically generated single-photons, we initialize the modes in the two interferometer arms in a state with density matrix $\rho_{\mathrm{om,out}}(p_{\mathrm{S}}, p_{\mathrm{aS}}, n_{\mathrm{write}}, n_{\mathrm{read}})$ obtained through the simulation procedure described above in Sec. H. Figure S9b shows the resulting second-order correlation function as a function of added thermal phonon occupancy during the readout pulse. The scattering probability during the write pulse ($p_{\mathrm{S}} = 0.1$) and initial phonon occupancy ($n_{\mathrm{write}} = 0.2$) due to heating from the write pulse are fixed at the values used in the HOM experiment presented in the main text. Again, the result of the QuTiP simulations agrees well with the experimental data point (indicated by a star in Fig. S9b) from the HOM measurement of optomechanically generated single photons as presented in the main text.

In Fig. S9b, the relatively high scattering probability of the write pulse leads to higher-order excitations of the mechanical mode during the two-mode squeezing interaction. Combined with the thermal phonon occupancy due to heating from the write pulse, this leads to deviations of the mechanical state before readout from the ideal single-phonon Fock state. Figure S9c shows the result of the same simulation for very low optomechanical scattering probability of the write pulse ($p_{\mathrm{S}} = 0.001$) and no initial thermal phonon occupancy $n_{\mathrm{write}} = 0$. For low added thermal phonon occupancy during the readout pulse, we obtain perfect HOM interference $g^{(2)}_{\mathrm{HOM}} = 0$.

Analytical model of HOM interference

We compare the results of our QuTiP simulations to a simple analytical model relating the value of $g^{(2)}_{\mathrm{HOM}}$ to the value of the auto-correlation function $g^{(2)}(0)$ as measured through an HBT experiment. We consider a general optical input state $\rho_{\mathrm{in}} = p_0 \left|0\right\rangle\left\langle 0\right| + p_1 \left|1\right\rangle\left\langle 1\right| + p_2 \left|2\right\rangle\left\langle 2\right| + \mathcal{O}(p_3)$. In an HBT measurement, assuming low click rates with $p_0 \approx 1 \gg p_1 \gg p_2$; the probability for a coincidence detection $p^{\mathrm{HBT}}_{\mathrm{c}}$ and for single click detection on detectors 1 and 2 $p^{\mathrm{HBT}}_{\mathrm{D1,D2}}$ approximated to second-order are then

$$p^{\mathrm{HBT}}_{\mathrm{c}} = \frac{1}{2} p_2 \tag{S18}$$

$$p^{\mathrm{HBT}}_{\mathrm{D1,D2}} = \frac{1}{2} p_1 + \frac{3}{4} p_2 \approx \frac{1}{2} p_1 . \tag{S19}$$

The value of the second order auto-correlation function is then obtained as

$$g^{(2)}(0) = \frac{p^{\mathrm{HBT}}_{\mathrm{c}}}{p^{\mathrm{HBT}}_{\mathrm{D1}} p^{\mathrm{HBT}}_{\mathrm{D2}}}, \tag{S20}$$

$$\approx \frac{2 p_2}{p_1^2} . \tag{S21}$$

In an HOM measurement, we analogously obtain

$$p_c^{\rm HOM} = p_2, \tag{S22}$$

$$p_{\rm D1,D2}^{\rm HOM} = p_1 + \frac{1}{2}p_1^2 + \frac{3}{8}p_2 \approx p_1, \tag{S23}$$

$$g^{(2)}_{\rm HOM} = \frac{p_c^{\rm HOM}}{p_{\rm D1}^{\rm HOM}p_{\rm D2}^{\rm HOM}}, \tag{S24}$$

$$= \frac{p_2}{p_1^2}. \tag{S25}$$

Finally, we can relate the value of the auto-correlation function $g^{(2)}(0)$ to the value of the HOM correlation function $g^{(2)}_{\rm HOM}$

$$g^{(2)}_{\rm HOM} = \frac{1}{2}g^{(2)}(0). \tag{S26}$$

For the simulations in Figs. S9b and c, we also simulate $g^{(2)}(0)$ of the state $\rho_{\rm om,out}$ and plot the result of Eq. (S26) as a solid line showing good agreement with the QuTiP simulation.

We observe in Fig. S9c that for the cross-polarized case and low added thermal phonon occupancy, the value of the HOM correlation function $g^{(2)}_{\rm HOM} < 1$. This observation is a result of low optical readout efficiency $\eta \ll 1$ and so large vacuum component in the optical states interfering at the beam splitter. We recover this result analytically by considering the optical input state at each port of the beam splitter approximated to first order $\rho_{\rm in} = p_0 \left|0\right\rangle\left\langle 0\right| + p_1\left|1\right\rangle\left\langle 1\right| + \mathcal{O}(p_2)$. For small p_2, we approximate $\rho_{\rm in} \approx (1-p_1)\left|0\right\rangle\left\langle 0\right| + p_1\left|1\right\rangle\left\langle 1\right|$. As above, we consider the coincidence and single click probabilities without approximating $p_0 \approx 1$ for now

$$p_c^{\rm HOM} = \frac{1}{2}p_1^2, \tag{S27}$$

$$p_{\rm D1,D2}^{\rm HOM} = \frac{3}{4}p_1^2 + p_1(1-p_1), \tag{S28}$$

$$g^{(2)}_{\rm HOM}(p_1) = \frac{p_c^{\rm HOM}}{p_{\rm D1}^{\rm HOM}p_{\rm D2}^{\rm HOM}}, \tag{S29}$$

$$= \frac{\frac{1}{2}p_1^2}{\left[\frac{3}{4}p_1^2 + p_1(1-p_1)\right]^2}. \tag{S30}$$

In the limit of high optical losses we obtain

$$\lim_{p_1\to 0} g^{(2)}_{\rm HOM}(p_1) = \frac{1}{2} \tag{S31}$$

in good agreement with the result obtained from the QuTiP simulation in Fig. S9c.

I. Single-photon and entanglement generation efficiency

Single-photon rate in the HBT measurement

The total efficiency $\eta_s = \eta_{\rm herald}\eta_{\rm conv}$ of our single-photon generation protocol during the HBT experiment is comprised of the efficiency of the phonon heralding ($\eta_{\rm herald}$) and the phonon-photon conversion process ($\eta_{\rm conv}$). The efficiency of the heralding process including the intrinsic optomechanical scattering probability of the Stokes scattering process $p_{\rm S} = 0.013$, optical cavity impedance ratio $\eta_{\rm cav} = \kappa_e/\kappa = 0.45$, lensed fiber coupling efficiency $\eta_{\rm fc} = 0.5$, efficiency of the optical filter setup $\eta_{\rm filters} = 0.4$, and single-photon detector efficiency (averaged over the two detectors) $\eta_{\rm SPD} = (\eta_{\rm SPD1} + \eta_{\rm SPD2})/2 = 0.58$ is then $\eta_{\rm herald} = p_{\rm S}\eta_{\rm cav}\eta_{\rm fc}\eta_{\rm filters}\eta_{\rm SPD} = 7\times 10^{-4}$. The efficiency of the conversion step with anti-Stokes scattering probability $p_{\rm aS} = 0.07$ is similarly obtained as $\eta_{\rm conv} = p_{\rm aS}\eta_{\rm cav}\eta_{\rm fc}\eta_{\rm filters}\eta_{\rm SPD} = 4\times 10^{-3}$. The corresponding rate of generated single photons with the repetition period of the pulse sequence $T_{\rm rep} = 10\,\mu$s (repetition rate $R_{\rm rep} = 100$ kHz) is $R_{\rm ph} = 0.23$ Hz.

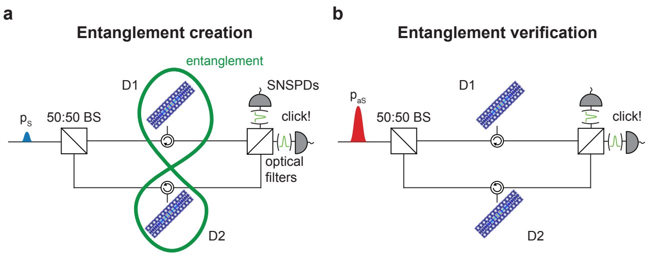

FIG. S10. **Protocol for remote entanglement generation and verification. a** Heralded creation of entanglement between two mechanical oscillators embedded in a phase-stabilized Mach-Zehnder interferometer. A blue-detuned pump pulse generates a phonon in one of the two mechanical modes. The second beam splitter erases the which-path information of the generated Stokes photons. Detection of a single photon at either of the two outputs of the interferometer projects the two mechanical modes into a maximally entangled state. **b** The created entangled state can be verified by readout of the mechanical states through a red-detuned readout pulse.

Projected entanglement rates

Remote entanglement between the mechanical modes of two OMCs can be created by embedding two such devices with matching mechanical and optical resonance frequencies into a phase-stabilized Mach-Zehnder interferometer [27]. This is an implementation of the Duan-Lukin-Cirac-Zoller (DLCZ) protocol for entanglement distribution in quantum networks [28]. By sending a blue-detuned pump pulse with Stokes scattering probability $p_{\rm S}$ into the interferometer and detecting a click on either of two single photon detectors at the two output ports of the interferometer, the joint state of the two mechanical modes is projected into a maximally entangled state $\left|\Psi\right\rangle = (\left|10\right\rangle + \left|01\right\rangle)/\sqrt{2}$. To verify the generated entanglement, a red-detuned readout pulse with anti-Stokes

scattering probability $p_{\rm aS}$ is sent into the interferometer. An entanglement witness can be defined for such an optomechanical entanglement experiment $R_{\rm m}$ [29], which can be expressed in terms of the cross-correlation functions between the Stokes and anti-Stokes click events as

$$R_{\rm m}(\theta,j)=4\frac{g^{(2)}_{{\rm r}_1,{\rm w}_j}(\theta)g^{(2)}_{{\rm r}_2,{\rm w}_j}(\theta)-1}{\left[g^{(2)}_{{\rm r}_1,{\rm w}_j}(\theta)-g^{(2)}_{{\rm r}_2,{\rm w}_j}(\theta)\right]^2}, \tag{S32}$$

where θ is a phase given by the relative phase between the two interferometer arms and the second-order cross-correlation functions between the write (Stokes field) and read (anti-Stokes field) pulses on the two detectors are defined as $g^{(2)}_{{\rm r}_i,{\rm w}_j} = \langle a^\dagger_{{\rm aS},i}a^\dagger_{{\rm S},j}a_{{\rm S},i}a_{{\rm aS},j}\rangle/(\langle a^\dagger_{{\rm aS},i}a_{{\rm aS},i}\rangle\langle a^\dagger_{{\rm S},j}a_{{\rm S},j}\rangle)$. A value $R_{\rm m}<1$ demonstrates entanglement of the two mechanical modes.

Thermal noise degrades the cross-correlation between the write and read pulse and leads to an increased value of $R_{\rm m}$. A theoretical analysis shows that, in general, a total thermal phonon occupancy during the read pulse of $n_{\rm th}<0.26$ is required to obtain $R_{\rm m}<1$ [29]. To achieve a statistically significant violation of this bound, previous demonstrations of remote entanglement between two one-dimensional nanobeam optomechanical crystals operated at reduced thermal phonon occupancy of $n_{\rm th}=0.12$ limiting the Stokes (anti-Stokes) scattering probabilities to $p_{\rm S}=0.007$ ($p_{\rm aS}=0.034$) [27]. Combined with the repetition period of the experiment $R_{\rm rep}=20\,$kHz and the total efficiency of the detection path $\eta=0.04$, entanglement of the two mechanical oscillators was heralded at a rate of $R_{\rm ent}=p_{\rm S}\eta R_{\rm rep}=6\,$Hz. Coincidence events between the write and read pulse required for verification of entanglement occurred at a rate of $R_{\rm coinc}=p_{\rm S}p_{\rm aS}\eta^2R_{\rm rep}=27\,{\rm h}^{-1}$.

Using our 2D OMC device, we can achieve similar levels of thermal noise with increased optomechanical scattering probabilities $p_{\rm S}=0.02$ ($p_{\rm aS}=0.16$). At these scattering probabilities the added thermal noise from the blue-detuned write pulse is $n_{\rm th,1}=0.043$ as calibrated from the measurements shown in Fig. S5. The red-detuned readout pulse adds an additional thermal noise of $n_{\rm th,2}=0.075$ as deduced from the thermometry measurement presented in the main text. The total added thermal noise is thus $n_{\rm tot}=0.118$. Combined with the repetition rate of our experiment $R_{\rm rep}=100\,$kHz and a total detection efficiency of $\eta=0.05$, we can project a heralded entanglement generation rate in a remote entanglement experiment of $R_{\rm ent}=100\,$Hz and a rate of coincidence events of $R_{\rm coinc}=2.9\times10^3\,{\rm h}^{-1}$ —an improvement by almost two orders of magnitude over previous demonstrations [27].

Through technical improvements to our optical setup by using adiabatically tapered fiber coupling with $\eta_{\rm fc}=0.85$ [30], using improved single-photon detectors with state-of-the-art detection efficiency $\eta_{\rm SPD}=0.9$, as well as straightforward adjustment of the cavity impedance ratio to operate in the ideal configuration of critical coupling $\eta_{\rm cav}=0.5$ (corresponding to a total detection path efficiency of $\eta=0.15$), the entanglement generation and verification rate can be boosted to $R_{\rm ent}=300\,$Hz and $R_{\rm coinc}=2.5\times10^4\,{\rm h}^{-1}$, respectively.

More elaborate improvements to the device geometry, such as using an evanescent coupling geometry [17], can be used to further reduce the thermal noise by a factor of six and thus enable higher entanglement rates by using larger optomechanical scattering rates. We can estimate the improvement to the entanglement rate attainable using such side-coupled 2D OMC devices by limiting the thermal noise to $n_{\rm th,max}=0.12$, the same level as in previous demonstrations of remote entanglement [27], and assuming fixed optomechanical phonon-photon conversion efficiency on the readout pulse for entanglement verification as above $p_{\rm aS}=0.16$. The thermal noise from the readout pulse would be reduced to $n_{\rm th,2}=0.013$, allowing a maximum added noise from the blue write pulse of $n_{\rm noise,1,max}=n_{\rm th,max}-n_{\rm th,2}=0.107$. We can estimate the added noise from the write pulse from the measurement results in Fig. S5 by dividing the thermal occupation from the prepulse by a factor of six to account for the reduction of thermal noise with an improved device geometry. The reduced thermal noise would allow for a substantial increase of Stokes scattering probability $p_{\rm S}$. Importantly, the thermal noise would be so low that the Stokes scattering probability is comparable or even larger than the added thermal noise from the prepulse. In this case, it is important to account for additional noise in the heralding process due to higher-order excitations from the two-mode squeezing interaction of the write pulse that are on the order of $p_{\rm S}$. The total noise originating write pulse is then $n_{\rm noise,1}=n_{\rm th,1}+p_{\rm S}$. We find that $n_{\rm noise,1}<n_{\rm noise,1,max}$ up to a Stokes scattering probability of $p_{\rm S}\approx0.08$ with a projected added thermal noise $n_{\rm th,1}\approx0.02$. This four-fold increase in Stokes scattering probability would translate to a four-fold increase in the entanglement rate $R_{\rm ent}=1.2\,$kHz.

We note that while the outcoupling efficiency of photons from our device can, in principle, be further increased by using an overcoupled optical cavity, the corresponding increase in total optical cavity linewidth κ would lead to a reduction in optomechanical scattering probability at the same intracavity photon number. For the pulse scheme used in this experiment, a critically coupled cavity provides the best trade-off between scattering probability and photon outcoupling efficiency [31].

Comparison to other quantum network platforms

We compare the projected entanglement rates attainable using 2D OMCs to the entanglement rates demon-

strated in recent state-of-the-art quantum network experiments. Firstly, we compare to an experiment where a single-photon interference scheme is used to generate remote entanglement between two nitrogen vacancy centers in diamond at a heralded entanglement rate of 39 Hz [32].

Secondly, we compare to an emissive quantum memory formed by an atomic ensemble [33]. In this work, the highest entanglement rate is achieved in an experiment in which two atomic quantum memories are connected by a 10 m long fiber and entanglement is heralded by a single-photon interference scheme. The experiment runs with a repetition time of $T_{\rm rep} = 20\,$ms out of which $T_{\rm load} = 18\,$ms are allocated to load and cool atoms and $T_{\rm ent} = 2\,$ms are allocated to perform entanglement trials with a repetition period of $\tau_{\rm ent} = 5\,\mu$s. The authors achieve an entanglement probability per trial of $P_{\rm ent} = 0.014$ yielding a total entanglement rate of $R_{\rm ent} = P_{\rm ent}T_{\rm ent}/(\tau_{\rm ent}T_{\rm rep}) = 280\,$Hz.

In both [32] and [33], the photons used for heralding the entanglement are not at telecom wavelength. For practical applications in remote quantum networks, wavelength conversion would be required and would lead to a substantial reduction in entanglement rate.

Lastly, we compare to an experiment in which single-photons generated by spontaneous parametric down-conversion are stored in atomic frequency comb quantum memories in solid-state crystals [34]. Entanglement between two quantum memories is established in a heralded fashion in a single-photon interference scheme in which the two memories are connected by 25 m of optical fiber. The authors achieve a record-high entanglement rate of 1.4 kHz.

[1] A. H. Safavi-Naeini, J. T. Hill, S. Meenehan, J. Chan, S. Gröblacher, and O. Painter, Two-dimensional phononic-photonic band gap optomechanical crystal cavity, Phys. Rev. Lett. **112**, 153603 (2014).

[2] H. Ren, M. H. Matheny, G. S. MacCabe, J. Luo, H. Pfeifer, M. Mirhosseini, and O. Painter, Two-dimensional optomechanical crystal cavity with high quantum cooperativity, Nat. Commun. **11**, 3373 (2020).

[3] C. M. Kersul, R. Benevides, F. Moraes, G. H. M. De Aguiar, A. Wallucks, S. Gröblacher, G. S. Wiederhecker, and T. P. Mayer Alegre, Silicon anisotropy in a bi-dimensional optomechanical cavity, APL Photonics **8**, 056112 (2023).

[4] F. M. Mayor, S. Malik, A. G. Primo, S. Gyger, W. Jiang, T. P. M. Alegre, and A. H. Safavi-Naeini, High photon-phonon pair generation rate in a two-dimensional optomechanical crystal, Nat. Commun. **16**, 2576 (2025).

[5] M. Vlassov, J. Pinto Moura, I. Marinkovic, A. Wallucks, N. Fiaschi, and S. Groeblacher, Filter cavity design by groeblacherlab (2022).

[6] S. Weis, R. Rivière, S. Deléglise, E. Gavartin, O. Arcizet, A. Schliesser, and T. J. Kippenberg, Optomechanically induced transparency, Science **330**, 1520 (2010).

[7] P. E. Barclay, K. Srinivasan, and O. Painter, Nonlinear response of silicon photonic crystal microresonators excited via an integrated waveguide and fiber taper, Opt. Express **13**, 801 (2005).

[8] J. Chan, T. P. M. Alegre, A. H. Safavi-Naeini, J. T. Hill, A. Krause, S. Gröblacher, M. Aspelmeyer, and O. Painter, Laser cooling of a nanomechanical oscillator into its quantum ground state, Nature **478**, 89 (2011).

[9] P. E. Barclay, K. Srinivasan, and O. Painter, Nonlinear response of silicon photonic crystal microresonators excited via an integrated waveguide and fiber taper, Opt. Express **13**, 801 (2005).

[10] S. M. Meenehan, J. D. Cohen, G. S. MacCabe, F. Marsili, M. D. Shaw, and O. Painter, Pulsed Excitation Dynamics of an Optomechanical Crystal Resonator near Its Quantum Ground State of Motion, Phys. Rev. X **5**, 041002 (2015).

[11] S. M. Meenehan, J. D. Cohen, S. Gröblacher, J. T. Hill, A. H. Safavi-Naeini, M. Aspelmeyer, and O. Painter, Silicon optomechanical crystal resonator at millikelvin temperatures, Phys. Rev. A **90**, 011803 (2014).

[12] H. Kobayashi, Y. Yamashita, T. Mori, Y. Nakato, T. Komeda, and Y. Nishioka, Interface states for si-based mos devices with an ultrathin oxide layer: X-ray photoelectron spectroscopic measurements under biases, Jpn. J. Appl. Phys. **34**, 959 (1995).

[13] Y. Yamashita, K. Namba, Y. Nakato, Y. Nishioka, and H. Kobayashi, Spectroscopic observation of interface states of ultrathin silicon oxide, J. Appl. Phys. **79**, 7051 (1996).

[14] T. Sakurai and T. Sugano, Theory of continuously distributed trap states at si-sio2 interfaces, J. Appl. Phys. **52**, 2889 (1981).

[15] G. P. Srivastava, *The Physics of Phonons*, 2nd ed. (CRC Press, Boca Raton, 2022).

[16] S. M. Meenehan, J. D. Cohen, S. Gröblacher, J. T. Hill, A. H. Safavi-Naeini, M. Aspelmeyer, and O. Painter, Silicon optomechanical crystal resonator at millikelvin temperatures, Phys. Rev. A **90**, 011803 (2014).

[17] S. Sonar, U. Hatipoglu, S. Meesala, D. P. Lake, H. Ren, and O. Painter, High-efficiency low-noise optomechanical crystal photon-phonon transducers, Optica **12**, 99 (2025).

[18] J. Guo and S. Gröblacher, Integrated optical-readout of a high-Q mechanical out-of-plane mode, Light Sci. Appl. **11**, 282 (2022).

[19] R. Riedinger, S. Hong, R. A. Norte, J. A. Slater, J. Shang, A. G. Krause, V. Anant, M. Aspelmeyer, and S. Gröblacher, Non-classical correlations between single photons and phonons from a mechanical oscillator, Nature **530**, 313 (2016).

[20] S. Hong, R. Riedinger, I. Marinković, A. Wallucks, S. G. Hofer, R. A. Norte, M. Aspelmeyer, and S. Gröblacher, Hanbury Brown and Twiss interferometry of single phonons from an optomechanical resonator, Science **358**, 203 (2017).

[21] M. J. Stevens, S. Glancy, S. W. Nam, and R. P. Mirin, Third-order antibunching from an imperfect single-photon source, Opt. Express **22**, 3244 (2014).

[22] L. Davidovich, Sub-Poissonian processes in quantum optics, Rev. Mod. Phys. **68**, 127 (1996).

[23] R. E. Fishman, R. N. Patel, D. A. Hopper, T.-Y. Huang, and L. C. Bassett, Photon-Emission-Correlation Spectroscopy as an Analytical Tool for Solid-State Quantum Defects, PRX Quantum **4**, 010202 (2023).

[24] J. R. Johansson, P. D. Nation, and F. Nori, QuTiP: An open-source Python framework for the dynamics of open quantum systems, Comput. Phys. Commun. **183**, 1760 (2012).

[25] J. R. Johansson, P. D. Nation, and F. Nori, QuTiP 2: A Python framework for the dynamics of open quantum systems, Comput. Phys. Commun. **184**, 1234 (2013).

[26] N. Fiaschi, B. Hensen, A. Wallucks, R. Benevides, J. Li, T. P. M. Alegre, and S. Gröblacher, Optomechanical quantum teleportation, Nat. Photon. **15**, 817 (2021).

[27] R. Riedinger, A. Wallucks, I. Marinković, C. Löschnauer, M. Aspelmeyer, S. Hong, and S. Gröblacher, Remote quantum entanglement between two micromechanical oscillators, Nature **556**, 473 (2018).

[28] L.-M. Duan, M. D. Lukin, J. I. Cirac, and P. Zoller, Long-distance quantum communication with atomic ensembles and linear optics, Nature **414**, 413 (2001).

[29] K. Børkje, A. Nunnenkamp, and S. M. Girvin, Proposal for Entangling Remote Micromechanical Oscillators via Optical Measurements, Phys. Rev. Lett. **107**, 123601 (2011).

[30] S. Gröblacher, J. T. Hill, A. H. Safavi-Naeini, J. Chan, and O. Painter, Highly efficient coupling from an optical fiber to a nanoscale silicon optomechanical cavity, Appl. Phys. Lett. **103**, 181104 (2013).

[31] R. Riedinger, *Single Phonon Quantum Optics*, Ph.D. thesis, University of Vienna (2018).

[32] P. C. Humphreys, N. Kalb, J. P. J. Morits, R. N. Schouten, R. F. L. Vermeulen, D. J. Twitchen, M. Markham, and R. Hanson, Deterministic delivery of remote entanglement on a quantum network, Nature **558**, 268 (2018).

[33] Y. Yu, F. Ma, X.-Y. Luo, B. Jing, P.-F. Sun, R.-Z. Fang, C.-W. Yang, H. Liu, M.-Y. Zheng, X.-P. Xie, W.-J. Zhang, L.-X. You, Z. Wang, T.-Y. Chen, Q. Zhang, X.-H. Bao, and J.-W. Pan, Entanglement of two quantum memories via fibres over dozens of kilometres, Nature **578**, 240 (2020), publisher: Springer Science and Business Media LLC.

[34] D. Lago-Rivera, S. Grandi, J. V. Rakonjac, A. Seri, and H. de Riedmatten, Telecom-heralded entanglement between multimode solid-state quantum memories, Nature **594**, 37 (2021).
